# Characterization of the *SWEET* Gene Family in Blueberry (*Vaccinium corymbosum* L.) and the Role of VcSWEET6 Related to Sugar Accumulation in Fruit Development

**DOI:** 10.3390/ijms26031055

**Published:** 2025-01-26

**Authors:** Jiaxin Liu, Xuxin Jiang, Lei Yang, Dongshuang Zhao, Yifei Wang, Yali Zhang, Haiyue Sun, Li Chen, Yadong Li

**Affiliations:** Engineering Center of Genetic Breeding and Innovative Utilization of Small Fruits of Jilin Province, College of Horticulture, Jilin Agricultural University, Changchun 130118, China; jiaxinl2002@163.com (J.L.); 18943165428@163.com (X.J.); 18943929275@163.com (L.Y.); zds1479@163.com (D.Z.); wangyifeisci@163.com (Y.W.); 17614413180@163.com (Y.Z.); haiyuesun@jlau.edu.cn (H.S.)

**Keywords:** blueberry, SWEET transporter, expression pattern, VcSWEET6, sugar accumulation

## Abstract

Sugars will eventually be exported transporters (SWEETs) are essential transmembrane proteins involved in plant growth, stress responses, and plant–pathogen interactions. Despite their importance, systematic studies on SWEETs in blueberries (*Vaccinium corymbosum* L.) are limited. Blueberries are recognized for their rapid growth and the significant impact of sugar content on fruit flavor, yet the role of the *SWEET* gene family in sugar accumulation during fruit development remains unclear. In this study, 23 *SWEET* genes were identified in blueberry, and their phylogenetic relationships, duplication events, gene structures, cis-regulatory elements, and expression profiles were systematically analyzed. The *VcSWEET* gene family was classified into four clades. Structural and motif analysis revealed conserved exon–intron organization within each clade. RT-qPCR analysis showed widespread expression of *VcSWEETs* across various tissues and developmental stages, correlating with promoter cis-elements. *VcSWEET6a*, in particular, was specifically expressed in fruit and showed reduced expression during fruit maturation. Subcellular localization indicated that VcSWEET6a is located in the endoplasmic reticulum. Functional assays in yeast confirmed its role in glucose and fructose uptake, with transport activity inhibited at higher sugar concentrations. Overexpression of *VcSWEET6a* in blueberries resulted in reduced sugar accumulation. These findings offer valuable insights into the role of *VcSWEETs* in blueberry sugar metabolism.

## 1. Introduction

Plant growth and development are influenced by numerous factors, with carbohydrates playing a pivotal role in the physiological processes of plants. As the primary form of carbohydrate, sugar serves not only as an energy source for plant growth and development but also actively participates in regulating gene expression, signal transduction, photosynthesis, and other crucial aspects of plant physiology [1]. Fruits contain a variety of sugars, including monosaccharides, oligosaccharides, and polysaccharides, which play a crucial role in determining quality traits such as color, taste, and aroma during the ripening process. Sugar transporters, which are categorized into H^+^-dependent and H^+^-independent transporters, play an important role in regulating sugar uptake, distribution, and storage, serving as crucial regulatory hubs within the sugar metabolism network in fruit [2,3]. SWEETs (Sugars will eventually be exported transporters), SUTs (Sucrose transporters), and MSTs (Monosaccharide transporters) are the main known sugar transporters in eukaryotes. Unlike SUTs and MSTs, H^+^-independent sugar transporter SWEET proteins mediate bidirectional cross-membrane movement of mono- and disaccharides by an alternating access mechanism [1,3,4]. The plant *SWEET* gene family is highly conserved, characterized by seven transmembrane domains (TMDs) and two MtN3/saliva domains that ensure proper function and stability [5]. OsSWEET2b is the first SWEET protein whose three-dimensional structure has been resolved. It features a pair of asymmetric triple helix bundles linked by a reverse-oriented transmembrane helix (TM4), which establishes the transport pathway. The phylogenetic analysis of the *SWEET* gene family reveals four distinct branches: Branch I is primarily involved in glucose transport, Branch II predominantly transports monosaccharides (hexoses), Branch III mainly facilitates sucrose transport, and Branch IV primarily regulates fructose transportation [5].

With the advancement of high-throughput sequencing technology, an increasing number of *SWEET* gene families have been identified in various plant species, including pineapple [6], grape [7], tomato [8], and apple [9]. However, systematic verification of their functions has only been conducted in model plants such as Arabidopsis and rice. For example, in Arabidopsis, AtSWEET2, AtSWEET4, AtSWEET11/12, AtSWEET15, and AtSWEET17 were all involved in sugar transport in the phloem. Among them, AtSWEET11/12 facilitated the transport of sucrose from leaves to vascular bundles. Under high light conditions, leaves of *atsweet11/12* mutant accumulated starch and sugars, exhibiting a phenotype of phloem loading deficiency [4]. *AtSWEET4* was highly expressed in leaf veins, overexpression of *AtSWEET4* led to an increase in plant size and a higher accumulation of glucose and fructose. In contrast, knocking down *AtSWEET4* resulted in smaller plants, reduced glucose and fructose content, chlorosis in the vein network, and decreased chlorophyll content in the leaves [10]. AtSWEET15 participated in seed development, root and leaf senescence, flower development, and stress resistance by regulating sugar transport and allocation [11,12,13]. Additionally, AtSWEET1, AtSWEET5, AtSWEET7-9, and AtSWEET13/14 participated in flower development. For instance, *AtSWEET1* was specifically expressed in anthers, *AtSWEET5* exhibits specific expression in pollen [14,15]. *AtSWEET8* participated in the deposition and polymerization of the primary exine, regulating pollen wall formation [16,17,18]. The expression of *AtSWEET13* and *AtSWEET14* was observed in the anther wall during the late stages of anther development, facilitating sucrose unloading into the locule. In the anthers of double mutants *atsweet13/14*, starch accumulated significantly, and sucrose unloading in the endothecium was obstructed, leading to reduced sucrose, fructose, and glucose content in pollen grains, which subsequently triggered pollen abortion due to poor development [19]. While AtSWEET9 played a crucial role in promoting nectar secretion [17]. Furthermore, SWEETs participated in responses to various biotic stress and abiotic stress. AtSWEET6 and AtSWEET10 have been identified to play essential roles in defense responses against pathogens. AtSWEET1 was involved in the interaction between nematodes and Arabidopsis and interacted with the Turnip mosaic virus P3 protein [20]. AtSWEET11 and AtSWEET12 enhanced Arabidopsis tolerance to low temperatures [21]. AtSWEET15 was induced by high salt, and its overexpression made plants more sensitive to salt stress and accelerated leaf senescence [13]. AtSWEET17 maintained cytoplasmic homeostasis by regulating fructose content in vacuoles in response to drought stress [22,23,24].

In the monocotyledonous model plant rice, SWEETs have also been identified to have multiple functions, particularly during biotic stress. For instance, OsSWEET11 could either promote or inhibit the growth of Xanthomonas oryzae pv. oryzae (Xoo), the causal agent of bacterial blight. Upon infection, Xoo secretes transcription activator-like effectors (TALEs), which subsequently bind to *cis-acting* elements in the promoter of SWEET genes, thereby regulating the expression of target *SWEET* genes and facilitating sugar transfer to Xoo. While the non-TALE effector XopN of Xoo contributes to the upregulation of *OsSWEET11* gene expression [12,25]. A recent study also found that the co-overexpression of *OsSWEET11a* and *OsSWEET14* modulated sucrose synthesis and defense responses to enhance immunity against bacterial blight in rice [26]. In addition, there were numerous reports on the involvement of OsSWEETs in seed filling. During rice caryopsis development, OsSWEET11/14/15, localized in the ovule and aleurone layer, promoted endosperm formation and complete filling. OsSWEET11/15 were responsible for sucrose transfer at the nucellus protrusion and the epidermis-aleurone layer interface during the milky stage [27,28,29]. *OsSWEET4*, with high-level expression in seeds, enhanced hexose transport to the endosperm, promoting sugar accumulation in grains and increasing seed filling rates [30,31]. The mutation *ossweet1b* induced carbon starvation in leaves, resulting in reduced sucrose, glucose, fructose, and starch contents during the filling process [32]. Although Arabidopsis and rice have made significant contributions as model plants in elucidating the function of the *SWEET* gene, it is imperative to conduct studies on other functions of SWEET in species exhibiting distinctive phenotypes. For instance, confirming the potential enhancement of seed oil and protein content by SWEET would be more compelling when investigated in soybean and *Xanthoceras sorbifolia* [33,34,35]. Similarly, it is crucial to explore plant materials that are more suitable for investigating sugar accumulation in fruits, which will further enhance our understanding of the mechanism underlying SWEET protein involvement in sugar transport processes.

Blueberry, a member of the *Vaccinium* genus in the Ericaceae family, is renowned for its delicate flesh, exceptional flavor, and rich nutrient content [36,37]. It is recognized as one of the five essential foods for human well-being by the FAO due to its manifold health benefits, encompassing anti-cancer, hypoglycemic, and anti-aging properties [38,39]. The commercial cultivation of blueberries originated in New Jersey in the northeastern United States in the early 20th century. Nowadays, blueberry cultivation can be found in 58 countries worldwide. By 2022, the global blueberry planting area had reached 256,701 hm^2^ with a yield increase to 1,734,696 t. The top ten blueberry-producing countries were China, the United States, Peru, Canada, Chile, Mexico, Poland, Morocco, Spain, and Portugal (the data were supported by the International Blueberry Organization). Despite China’s prominent position in terms of blueberry cultivation area and yield, the limited commercialization rate of fresh blueberries hinders the sustainable development of China’s blueberry industry due to extensive cultivation of processed varieties, outdated fresh varieties, and inadequate cultivation management. The sugar components present in blueberry fruit consist of glucose, fructose, and sucrose, exerting a direct influence on the flavor profile of blueberries. However, the process and mechanism of sugar accumulation in blueberry fruit remain poorly understood. The recent publication of the blueberry genome sequence has provided a fundamental resource for investigating the *SWEET* gene family in blueberry [40]. In this study, we identified 23 *SWEET* gene members and analyzed their phylogenetic relationships, duplication events, conserved motifs, promoter elements, and expression patterns. Additionally, we also cloned the gene *VcSWEET6a*, which is specifically expressed during the early stages of fruit development, and investigated its function. The results of this study provide comprehensive information for understanding VcSWEETs and provide valuable insights into sugar accumulation during blueberry fruit development and ripening.

## 2. Results

### 2.1. Genome-Wide Identification of SWEETs in Blueberry

The *SWEET* genes were identified through BLAST searches against the COGE database, followed by the removal of redundant sequences. A total of 23 *VcSWEET* genes with confirmed conserved domains were identified from the blueberry genome and named according to Doidy’s taxonomic framework [41]. The physicochemical properties of *VcSWEET* genes are summarized in Supplemental Table S1. These *VcSWEETs* encoded proteins ranged from 180 aa to 433 aa with molecular weight from 20.13 and 47.94 kDa and theoretical isoelectric points from 8.44 to 9.81. The hydropathicity (GRAVY) values of VcSWEETs ranged from 0.152 to 0.978, while the instability index varied between 29.53 and 50.09, indicating that all VcSWEETs were characterized as hydrophobic proteins with approximately 70% being classified as stable proteins. The majority of VcSWEETs were predicted to possess seven transmembrane regions, which represent conserved domains shared by SWEET proteins. However, a minority exhibited only 5 or 6 transmembrane regions due to incomplete splicing. The results of subcellular localization prediction showed that all VcSWEETs were localized to membrane structures. Specifically, four members were found in the tonoplast membrane (TM), while 18 members were observed in the plasma membrane (PM). Notably, one member was exclusively detected in the chloroplast membrane (CHL).

### 2.2. Phylogenetic Analysis, Chromosomal Location, and Synteny Analysis

**Figure 1:**
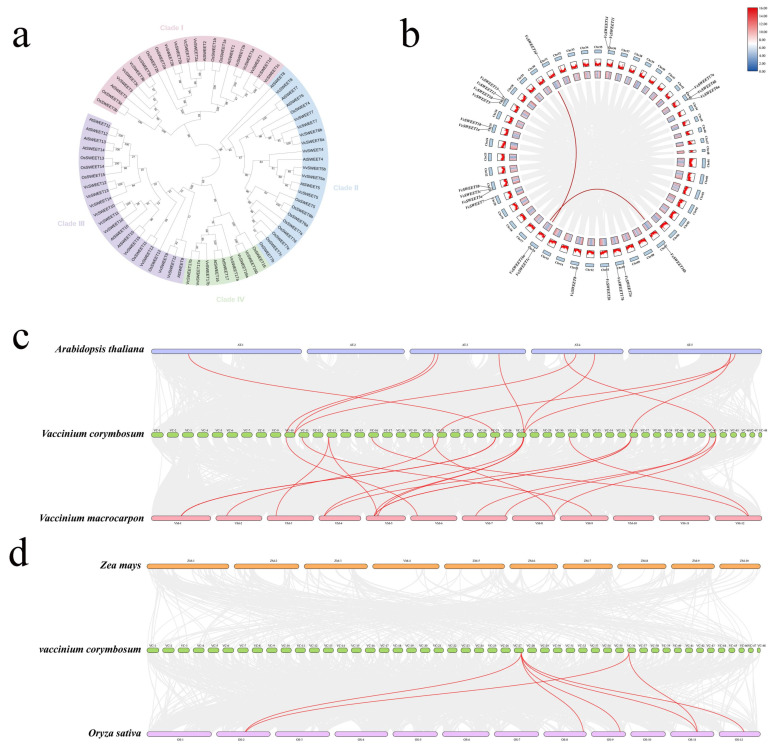
Polygenetic relationship and synteny analysis of *VcSWEETs* from blueberry: (**a**) Phylogenetic tree of the *SWEET* gene family in blueberry (*Vaccinium corymbosum* L.), rice (*Oryza sativa*), Arabidopsis (*Arabidopsis thaliana* L.), and grape (*Vitis vinifera* L.). (**b**) Synteny analysis of *VcSWEETs* in blueberry. (**c**,**d**) Synteny analysis of *VcSWEET* genes between blueberry and Arabidopsis (*Arabidopsis thaliana* L.), cranberry (*Arabidopsis thaliana* L.), corn (*Zea mays* L.), and rice(*Oryza sativa*). Gray lines in the background indicated the syntenic blocks within blueberry and other plant genomes. The highlighted color represented *VcSWEETs* with synteny in different genomes.

The evolutionary relationships among *SWEET* genes were investigated by constructing a neighbor-joining phylogenetic tree, which involved aligning VcSWEET sequences with AtSWEETs, OsSWEETs, and VvSWEETs (Figure 1a). The results revealed that all VcSWEET members were classified into four distinct clades: Clade I comprised 9 VcSWEETs, 3 AtSWEETs, 6 OsSWEETs, and 2 VvSWEETs; Clade II contained 5 VcSWEETs, 5 AtSWEETs, 9 OsSWEETS and 4 VvSWEEts; Clade III consisted of 5 VcSWEETs, 7 AtSWEETs, 5 OsSWEETs, and 5 VvSWEETs; and Clade IV included 4 VcSWEETs, 2 AtSWEETs, 1 OsSWEETs, and 3 VvSWEETs.

A gene family is a group of several similar genes that originate from the duplication event of a single ancestral gene and typically exhibit analogous biochemical functions. Gene replication events include segmental and tandem duplications. Tandem replication predominantly occurs within the region of chromosome recombination. According to previous descriptions, a chromosomal region spanning 50 kb and encompassing two or more genes is defined as a tandem duplication event [42]. This study demonstrated an uneven distribution of *VcSWEETs* across the 12 chromosomes (Figure 1b). Chromosome 27 harbored the highest number of *SWEET* genes, with four *VcSWEETs* present, followed by chromosomes 21 and 43, each containing three *VcSWEETs*. The *VcSWEET* genes were distributed across chromosomes 10, 25, 16, and 36 in duplicate, while a single *VcSWEET* gene was present on chromosomes 7, 11, 13, 20, and 31. Notably, the genomic distance between *VcSWEET3a*, *3b*, and *3c* on chromosome 21, *VcSWEET6a* and *6b* on chromosome 43, *VcSWEET10*, *12*, and *13* on chromosome 27, as well as *VcSWEET11*, and *14* on chromosome 36, was found to be remarkably short, indicating the presence of tandem duplications in chromosomes 21, 27, 36, and 43.

The occurrence of segmental duplication resulted in the replication of genes that were widely separated and even situated on different chromosomes. The genes generated by the large fragment duplication exhibit a high degree of similarity to their neighboring genes, so thus MCScan was frequently employed for analyzing segmental duplication in gene families with large replication fragments. The results displayed segmental duplications that were found on chromosomes 21, 25, and 43. These results indicated that the distribution of genes was diversiform across different chromosomes. We analyzed the mechanisms of *VcSWEETs* expansion and evolution. Only two gene pairs, *VcSWEET1c*/*VcSWEET1d* and *VcSWEET16a*/*VcSWEET16b*, participated in gene duplication events by segmental repeats. It followed that segmental repeats were the main causes of *VcSWEETs* expansion. The syntenic gene pairs between species were analyzed (Figure 1c,d). Blueberry had 15 syntenic gene pairs with cranberry, but had 7 with Arabidopsis. However, we found fewer syntenic gene pairs with the monocots maize and rice, four pairs with rice, but none with maize. The conservatism between blueberry and cranberry was relatively high, while the variability with Arabidopsis, maize, and rice was greater. We also found that the homologous genes of *VcSWEET10*/*14* exist in both dicotyledonous and monocotyledonous plants, indicating that these genes were formed before species differentiation.

### 2.3. Analysis of Conserved Motif and Gene Structure of VcSWEETs

**Figure 2:**
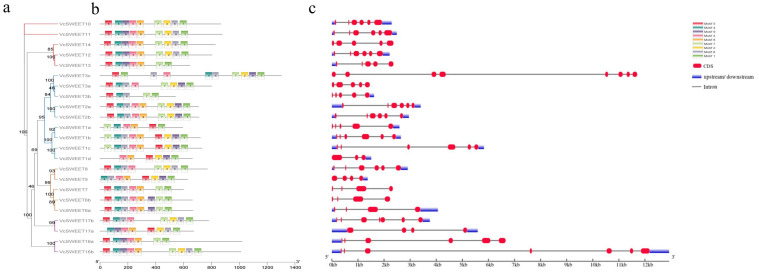
Phylogenetic relationships, conserved motif composition, and gene structure of 23 *VcSWEETs*: (**a**) Phylogenetic tree. (**b**) Motif pattern of *VcSWEETs*. The 8 colored boxes represent 8 different motifs, and their positions represent the positions on the protein. (**c**) Gene structure. CDS and introns are represented by red rectangles and black single lines, respectively.

A total of nine conserved motifs were identified among *VcSWEETs* using the MEME (Version 5.5.7) tool (Figure 2b). Motif 1 and motif 5 were found to be present in all members of *VcSWEETs*, with the exception of *VcSWEET1d*, which lacked Motif 4. The *VcSWEET3b* gene exhibited the lowest motif count, as it lacked Motif 2, Motif 3, Motif 8, and Motif 9. Two *VcSWEETs* were found to lack Motif 7, while four *VcSWEETs* were observed to lack Motif 6. Additionally, the majority of *VcSWEETs* within the same clade exhibited similar motif arrangements, indicating robust structural conservation and functional similarity among these genes. The arrangement of introns and exons was analyzed to gain a deeper understanding of the structural differences in *VcSWEET* genes. As shown in Figure 2c, the 23 *VcSWEETs* exhibited a range of 3–8 exons, with the corresponding number of introns ranging from 2 to 7. The variation in intron numbers across different branches was not obvious. In Clade I, *VcSWEET* genes exhibit a range of 2–7 introns, while in Clade II the range was 3–5, in Clade III it was 4–5, and in Clade IV it was 3–6. Furthermore, within Clade II, all *VcSWEETs* exhibited the same exon count of four, except for *VcSWEET8*, which had six.

### 2.4. Cis-Acting Elements Analysis of VcSWEET Gene Family

**Figure 3:**
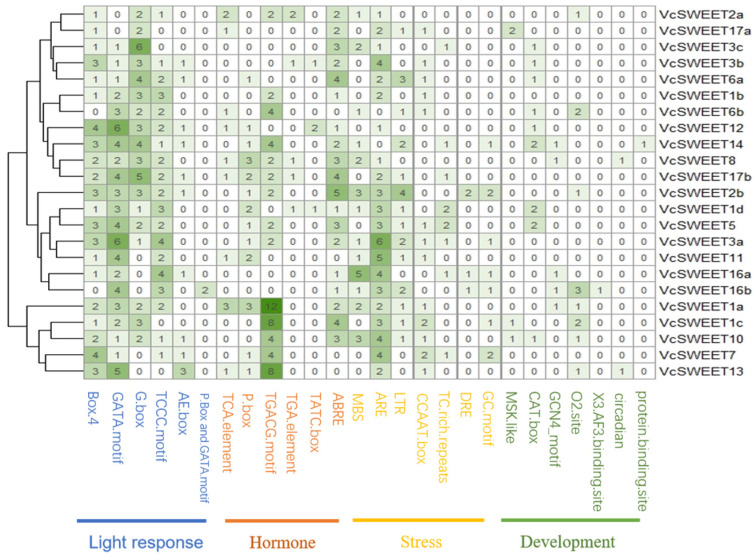
Prediction of *cis-acting* elements in the promoters of *VcSWEETs*. The gradient color in the cell represents the number.

The potential regulatory factors of *VcSWEET* genes were investigated by predicting promoter *cis-acting* elements using PlantCARE (Figure 3). The analysis revealed the presence of 25 *cis-acting* elements within the *VcSWEET* genes family, which can be categorized into four main groups: light-responsive elements, hormone-related elements, abiotic stress-related elements, and growth and development-related elements. The light response elements included Box 4, AE-box, TCCC-motif, G-Box, and GATA-motif. The GATA-motif was the most numerous light response element (62), followed by Box 4 (43). Seven hormone-responsive *cis-acting* elements were identified. The TGACG-motif and TCA-element represented two distinct types of JA-responsive *cis-acting* elements, with the former exhibiting the highest abundance of hormone-response elements. Notably, VcSWEET1a contained 12 TGACG-motif and 3 TCA-element within its promoter sequence, which may play a pivotally important role in pathogen–host interactions. Most *VcSWEETs* were related to the ABA pathway, and 19 *VcSWEETs* promoters contained 46 ABRE. We also identified 22 P-box, 4 TATC-box associated with the GA_3_ pathway, and 6 TGA-element related to the IAA pathway in *VcSWEETs*. Some stress-responsive *cis-acting* elements were also found in promoters. The most numerous stress-responsive elements were ARE (58), followed by the drought-induced elements MBS (24) and low temperature-induced elements TLR (22). The number of growth and development-related elements was fewer, which were related to the regulation of zein metabolism (O2-site), meristem specificity (CAT-box), endosperm expression (GCN4-motif), and so on.

### 2.5. Expression Pattern of VcSWEET Genes in Different Organs and Fruits at Four Developmental Stages

**Figure 4:**
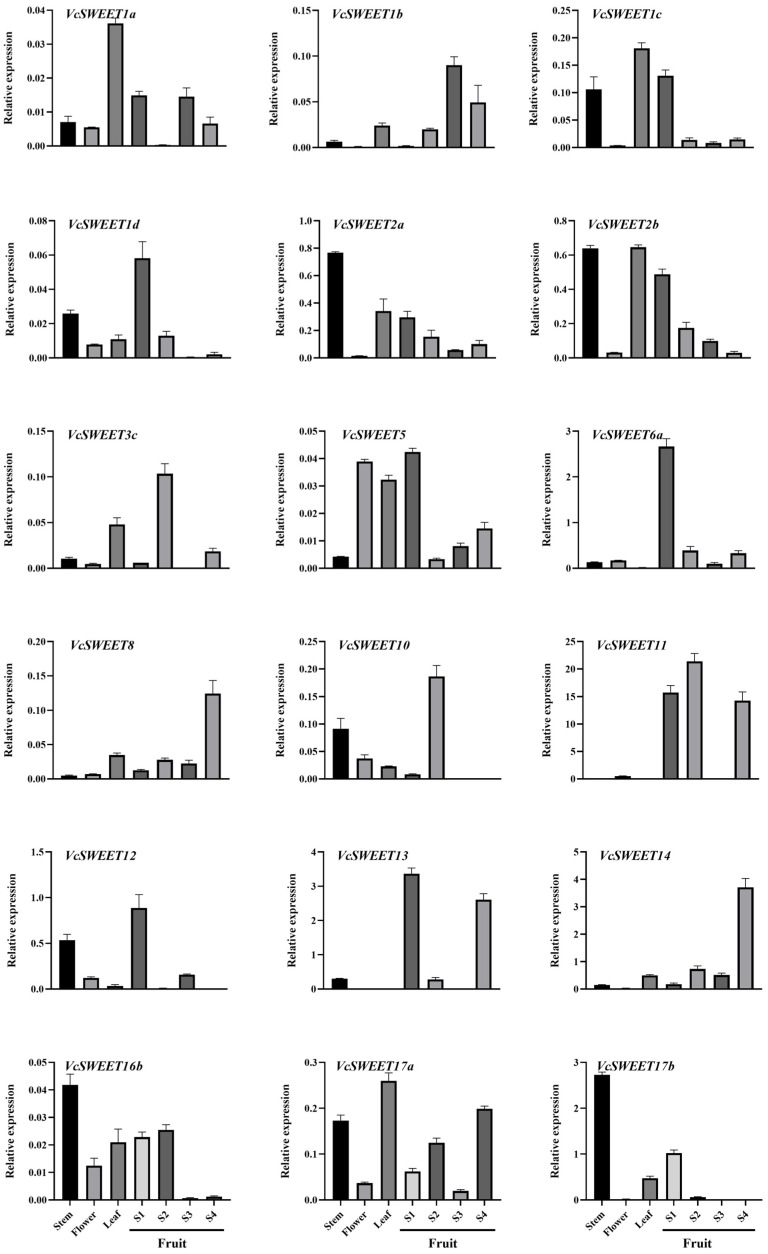
Expression pattern of *VcSWEETs* during different organizations and during fruit development periods.

To explore the potential roles of *VcSWEETs* in various organs of blueberry, RT-qPCR analysis was conducted on leaves, stems, flowers, and fruits at four distinct developmental stages (Figure 4). Among the 23 *VcSWEET* genes, 18 exhibited expression in diverse organs and developmental stages of fruits, albeit displaying distinct patterns of expression. The expression level of *VcSWEET17b* was found to be the highest among all gene family members in stems, followed by *VcSWEET2a*, *VcSWEET2b*, and *VcSWEET16b*. The genes *VcSWEET1a*, *VcSWEET2b*, and *VcSWEET17a* exhibited specific expression patterns in leaves, with the highest expression level observed for *VcSWEET17a* among the three genes. And *VcSWEET5* was expressed specifically in flowers. Additionally, the expression of 10 genes in fruit was specific; however, their patterns of expression vary throughout fruit development. The expression levels of *VcSWEET8* and *14* increased progressively during fruit development. Conversely, the expression of *VcSWEET1d*, *5*, *6a*, *12*, and *13* reached its peak in the early stage of fruit development. *VcSWEET1b*, *3c*, and *10* exhibited maximal expression during the middle stage of fruit development with a notable peak observed for *VcSWEET3c* and *10* during the S2 period while the peak value for *VcSWEET1b* occurred during the S3 period.

### 2.6. Clone and Subcellular Localization of VcSWEET6a

By comparing the expression of *VcSWEET6a*, sugar content, and the activity of sucrose-metabolizing enzymes at different fruit developmental stages (Supplemental Figure S1), we observed a negative correlation between the expression level of *VcSWEET6a* and both sugar content and SS enzyme activity. Therefore, we speculated that the role of *VcSWEET6a* was related to sugar accumulation in blueberry fruit development. Subsequently, we cloned the CDS region of *VcSWEET6a* and successfully obtained a band with a total length of 787 bp. To further investigate the conserved domains of VcSWEET6a, it was aligned with five other SWEET proteins. The alignment revealed that in VcSWEET6a, the amino acid residue L25 (proline) was substituted with M (methionine), and T75 (threonine) was replaced by I (isoleucine) in THB1. Notably, similar mutations were also present in THB2, where L154 was replaced by M. Additionally, the presence of base deletions in TM5 of SWEET6a. That may imply potential neofunctionalization or subfunctionalization (Supplemental Figure S2).

**Figure 5:**
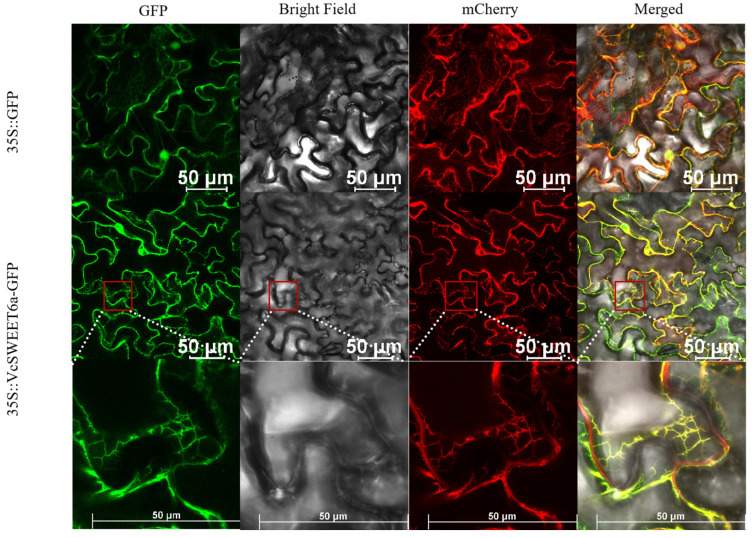
Subcellular location analysis of VcSWEET6a was performed in *N. benthamiana* leaf. The free GFP was the control. Red fluorescence represents the control.

Subcellular localization prediction using the WOLF PSORT II tool showed that VcSWEET6a was located on the plasma membrane. In order to precisely determine the subcellular localization, we transiently expressed a fusion protein of VcSWEET6a and GFP in tobacco leaves. The result of co-expression observed by confocal laser scanning microscopy did not show a complete overlap between the red fluorescence signal from the plasma membrane marker and the green fluorescence signal from VcSWEET6a-GFP (Supplemental Figure S3). Subsequently, an ER marker was used to co-express with VcSWEET6a-GFP, and we found that the GFP signals from the fusion protein were co-localized with the endoplasmic reticulum marker (Figure 5), suggesting that VcSWEET6a is an ER-located protein.

### 2.7. VcSWEET6a Transports Hexose in Yeast

**Figure 6:**
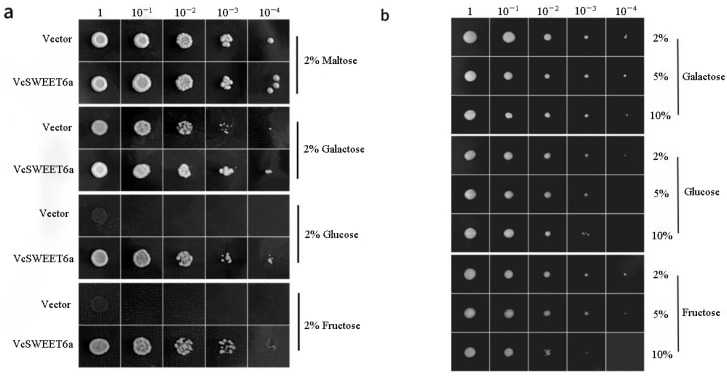
Yeast growth of VcSWEET6a expressed in yeast (Saccharomyces cerevisiae). Yeast growth assay of *EBY.VW4000* transformed with pDR196 and pDR196-VcSWEET6a on 2% maltose, galactose, glucose, and fructose: (**a**) Yeast growth under different carbon sources; (**b**) The growth of yeast under different sugar concentrations.

To characterize the transport characteristics of *VcSWEET6a*, we heterologously expressed the CDS of *VcSWEET6a* using the yeast expression vector pDR196 in the EBY.VW4000 yeast strain, while employing empty vector pDR196 as negative controls (Figure 6a). The EBY.VW4000 strain can only grow on selective media with maltose as the sole carbon source. Results demonstrated that all treatments exhibited growth on maltose without distinction. However, EBY.VW4000 transformed with empty vector pDR196 failed to grow on 2% (*w*/*v*) sucrose, whereas expression of *VcSWEET6a* enabled its growth on glucose and fructose. These findings provided evidence for the ability of VcSWEET6a to transport hexose in yeast. Additionally, three sugar concentration gradients were set at 2%, 5%, and 10% in the medium. The results indicated a significant decrease in the transport efficiency of VcSWEET6a with increasing sugar concentration, suggesting its dependence on sugar concentration (Figure 6b).

### 2.8. Transient Overexpression of VcSWEET6a

**Figure 7:**
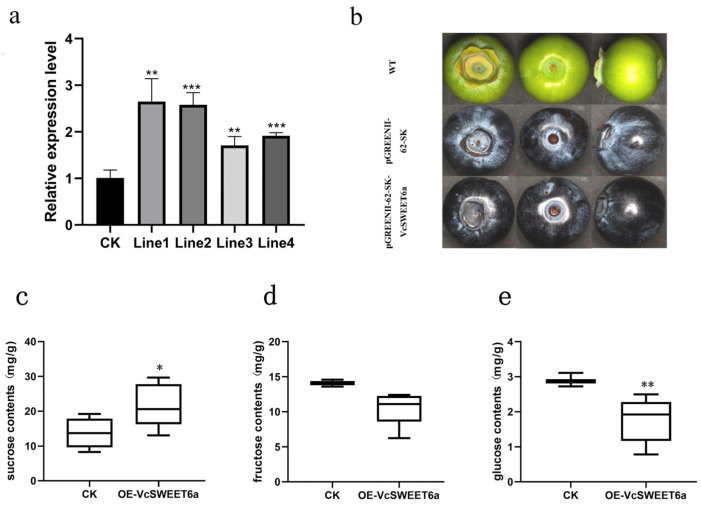
Overexpression of *VcSWEET6a* enhances sucrose accumulation in transgenic blueberry fruits: (**a**) Expression of VcSWEET6a in different overexpressed lines. (**b**) Images of wild-type (WT), pGREENII-62-SK-overexpression, and *VcSWEET6a*-overexpression blueberry. (**c**) The content of sucrose. (**d**) The content of fructose. (**e**) The content of glucose. “*” indicates significant differences at the 0.05 level, and “**” indicates significant differences at the 0.01 level. “***” indicates significant differences at the 0.001 level.

We transiently overexpressed *VcSWEET6a* in blueberry fruit at the expansion stage to validate its function. The results showed that the overexpression fruit exhibited accelerated development compared to the wild-type, with a significant upregulation of *VcSWSEET6a* expression by up to threefold in the successfully overexpressed fruits (Figure 7a,b). Moreover, the content of glucose and fructosein in overexpressed fruits exhibited a decrease compared to the control, with reductions of 23% and 13%, respectively. Interestingly, the content of sucrose was significantly increased by 3.6 times (Figure 7c–e)

## 3. Discussion

### 3.1. Evolutionary Analyses of VcSWEET Genes

SWEET transporter is widely found in eukaryotic unicellular organisms, higher plants, and animals. *C. elegans* has seven SWEET genes, Drosophila possesses two *SWEET* genes, whereas humans have only one [1,4]. The number of *SWEET* genes in plant genomes exceeds that in animals. In general, angiosperm genomes have about 15–25 *SWEET* genes, such as grape (17) [43], pomegranate (20) [44], sorghum (23) [45], and mulberry (24) [46]. Based on the available reports, we observed that the number of *SWEETs* within Angiosperms can vary significantly, ranging from a minimum of 7 in loquat to a maximum of 108 in wheat [38,47]. The variation in gene family scale between different species can be ascribed to gene duplication events, which play a crucial role in the evolution of gene families [48]. Marivi, et al. discovered that tandem duplications may have greatly contributed to the metabolic diversity observed in blueberry [40]. In this study, we identified 23 *SWEET* genes in the blueberry genome, which exhibited an uneven distribution across 12 chromosomes. Among these, *VcSWEET3a/3b/3c*, *VcSWEET6a/6b*, *VcSWEET10/12/13*, and *VcSWEET11/14* were considered descendant genes derived from tandem duplications. In addition, the gene pairs *VcSWEET1c/1d* and *VcSWEET16a/16b* had undergone gene duplication events through segmental repeats (Figure 1b). Therefore, we speculated that the expansion of the *VcSWEET* gene family in blueberry was facilitated by both segmental tandem duplications and segmental duplications during the course of evolution. During evolution, duplicated genes may experience different fates: some may lose function and ultimately be removed through deletion, while others may persist and evolve different functions [49]. Previous research has confirmed that in Arabidopsis, rice, soybean, and ginkgo, half or more of the duplicated genes exhibit functional divergence and differential expression [50]. More than half of the duplicated *VcSWEET* genes also exhibited differential expression (Figure 4), implying significant functional divergence in duplicated *VcSWEET* gene pairs.

Furthermore, we conducted an analysis of syntenic gene pairs across species. Blueberry exhibited 15 syntenic gene pairs with cranberry and 7 with Arabidopsis. However, the number of syntenic gene pairs between blueberry and the monocots corn and rice was relatively lower, with only four pairs identified with rice and none found with corn. It was intriguing that orthologous genes of *VcSWEET2a/3a/10/12/13* were identified across all dicots, while being conspicuously absent in monocots, implying their emergence subsequent to species divergence. The syntenic gene pairs between blueberry and cranberry were more abundant compared to those between blueberry and Arabidopsis, suggesting *SWEET* families in blueberry and cranberry might share a common ancestor and similar evolutionary patterns to adapt to the environment. Recent genome analysis confirmed that the Ericaceae plants had undergone two whole-genome duplications, the ancient γ-triplication and a more recent whole-genome duplication, which were shared with cranberry, blueberry, and rhododendron [51,52]. Comparative genomics within the *Vaccinium* genus suggested cranberry divergence occurred 4.5 Mya, following their divergence from blueberry 10.4 Mya. However, compared with blueberry, cranberry has not suffered more recent whole-genome duplications [53]. This can also be confirmed by examining the size of the *SWEET* gene family, which reveals a twofold increase in blueberry *SWEET* family members compared to that observed in cranberry. We speculated that during the evolution of blueberry, the *SWEET* gene family expanded, with gene duplication leading to neofunctionalization or subfunctionalization, such as an increased sugar accumulation and a more intense flavor in fruits. The prediction of *cis-acting* elements in the upstream sequences of *VcSWEET* genes also indicated that VcSWEET proteins were likely involved in phytohormone signaling pathways and abiotic stress responses.

### 3.2. The Function Predication of VcSWEET Genes Based on Spatial and Temporal-Specific Expression Analysis

The differential expression of gene family members across various tissues and conditions reflected the functional diversity inherent in their roles, which can be attributed to either functional specialization or redundancy developed during evolutionary adaptation to the environment. Out of the 23 *VcSWEET* genes, eighteen genes were expressed in different developmental stages of fruits and various organs, albeit with distinct expression patterns. In stems, *VcSWEET2a* and *VcSWEET17b* exhibited high expression levels. So far, the role of *SWEET* in stems has only been documented in some plants where the stem serves as the primary storage organ. In sugarcane, the expression of *SsSWEET2a*, which facilitated sucrose efflux from source to sink tissues, was predominantly observed in immature stems, whereas the specific expression of *SsSWEET13c* during maturation and maturity stages of the stems contributes to sugar accumulation [54]. The stems of blueberries did not function as the primary storage organs; therefore, it was hypothesized that *VcSWEET2a* and *VcSWEET17b* may exhibit analogous functionalities to *SsWEET2a*, facilitating sucrose efflux from source to sink tissues instead of sugar accumulation in the stem. In source organ leaves, both *VcSWEET1a* and *VcSWEET17a* showed high expression. Similarly, the homologous gene *VvSWEET1* showed a comparable expression pattern to *VcSWEET1a*, primarily observed in young and mature grape leaves [55,56]. *AtSWEET11* and *AtSWEET12* displayed high expression levels in Arabidopsis leaves and played crucial roles in the phloem loading of sucrose from source leaves [4]. Additionally, the absence of *OsSWEET1b* another homolog of *VcSWEET1a*, expedited sugar deprivation and consequently accelerates rice leaf senescence [57]. Furthermore, *MdSWEET4* exhibited abundant expression in the young leaves of strawberry. Overexpression of *MdSWEET4* in apple plants not only promoted seed germination but also accelerated flowering progression and enhanced fruit ripening [58]. So, we speculated that *VcSWEET1a* and *VcSWEET17a* might play a role in the phloem loading of assimilates in the source leaf, thereby potentially impacting the growth and development of other skin organs.

The processes of phloem unloading and post-phloem transport play a crucial role in regulating sugar distribution among storage organs and maintaining fruit’s storage strength, which significantly impacts fruit yield and quality. The gene expression within *VcSWEETs* exhibited significant divergence between vegetative and reproductive organs, with a predominant enrichment of genes in the latter. Among reproductive organs, only *VcSWEET5* showed high expression in flowers, while 10 other *VcSWEETs* exhibited specific expression in fruits. At present, several studies have reported the role of SWEET in regulating sugar transport and accumulation within fruits. For example, *CsSWEET7a/b* and *15*, which were involved in the regulation of sugar unloading processes, exhibited a high level of expression and transcriptional abundance in mature cucumber fruit [35,59]. In apples, *MdSWEET23* demonstrated elevated expression levels during fruit maturation, and its suppression led to reduced sucrose accumulation in the fruit, thereby affecting sorbitol transport and metabolism [58]. Similarly, *PbSWEET5* in pears displayed comparable expression patterns that facilitate sucrose transport and promoted fruit development [60]. In our study, the expression of *VcSWEET8* and *VcSWEET14* was upregulated during fruit maturation; conversely, the expression of *VcSWEET1d*, *5*, *6a*, *12*, and *13* was downregulated. Additionally, maximal expression of *VcSWEET1b*, *3c*, and *10* was observed during the intermediate stage of fruit development. So, we speculated that the 10 *VcSWEETs* were involved in the development and ripening of blueberry fruits. However, further validation was required to determine whether they act synergistically or exhibit functional redundancy.

### 3.3. The ER-Located VcSWEET6 Negatively Regulates the Accumulation of Hexose in Blueberry Fruit

In response to their diverse functions, the localization of SWEET proteins also exhibits diversity. According to the current report, the majority of SWEET proteins are predominantly localized in the plasma membrane or vacuolar membrane, with only a limited presence observed in other cellular membranes. In Arabidopsis, AtSWEET1, 4, 8, and all members of Clade III (9–15) were localized on the plasma membrane, facilitating sugar transport across this cellular barrier. Additional research found that AtSWEET9, which was involved in nectar secretion, also exists on the trans-Golgi network (TGN) of the Golgi apparatus [17]. The TGN serves as the exit region of the Golgi apparatus, playing a crucial role not only in the sorting, packaging, and export of proteins but also in intracellular sugar synthesis. Sugars can be transported into and out of the vacuole through sugar transporters located on the vacuolar membrane. Once inside, sugars have the ability to accumulate, creating a highly concentrated sugar environment within the vacuole. This elevated concentration not only serves as an energy storage mechanism but also functions as an osmoregulator, aiding in cellular water balance regulation. When energy is required by the cell, specific transport proteins facilitate the release of sugars from the vacuole into the cytoplasm. The nature of this process can either be active or passive depending on the concentration gradient of sugars between both sides of the cell. AtSWEET2, 16, and 17 were localized on the vacuolar membrane, corresponding to their function of transporting sugars in the vacuole, indicating a certain correlation between localization and function [22,23]. In our study, VcSWEET6a was found to be localized on the ER membrane, which is consistent with its homologs AtSWEET6 [1]. It is widely known that ER is capable of synthesizing proteins and lipids, and it is also associated with carbohydrate metabolism. In particular, the smooth endoplasmic reticulum is related to sugar synthesis. Previous research has shown that ER-localized sugar transporters were associated with sugar accumulation. For example, the overexpression of the hexose transporter AtSTP8 led to an elevation of hexose concentrations in Arabidopsis leaves [61]. Additionally, the OsSAC1 protein, encoded by the Sugar Accumulation1 gene, was also found to be localized in the ER. The ossac1 mutation resulted in sugar accumulation in rice leaves [62]. VcSWEET6a belonged to Clade II, whose members have the function of transporting hexose. Therefore, it was speculated that VcSWEET6a may be involved in the accumulation of hexose in blueberry fruits. To verify the hexose transport function of VcSWEET6a, we conducted yeast substrate specificity expression analysis and found that the VcSWEET6a protein can transport glucose and fructose, consistent with the characteristics of Clade II. At the same time, it was also found that as sugar concentration increases, the sugar transport activity of VcSWEET6a was inhibited. The transport capacity of sugar transporters may exhibit saturation. As the intracellular sugar concentration increases, sugar transporters may reach their maximum transport capacity, resulting in a failure of the transport rate to increase further, and possibly even a decrease. Alternatively, the activity of VcSWEET6a may be inhibited by a feedback mechanism when intracellular sugar concentrations are elevated.

The crucial role of *SWEET* genes in regulating sugar accumulation in fruits has been demonstrated in various plants. In jujube, the expression of *ZjSWEET11* and *ZjSWEET18* gradually increased during fruit development, peaking at complete maturity [63]. In pineapple, *AcSWEET11* was strongly expressed in ripening fruits, and the overexpression of *AcSWEET11* in the pineapple callus and in tomato enhanced sugar content [64]. In grapes, *VvSWEET10* was strongly expressed at véraison, and overexpression in grapevine calli and tomatoes significantly increased the content of glucose, fructose, and total sugar [65]. In contrast to the positively regulated mechanism, our study demonstrated a decline in the expression level of *VcSWEET6a* alongside a gradual increase in sugar content during fruit maturation. Transient overexpression of *VcSWEET6a* in blueberry fruits leads to a significant decrease in fructose and glucose contents, accompanied by a marked increase in sucrose content, suggesting that the expression of *VcSWEET6a* negatively regulates sugar accumulation in blueberry fruits. This is consistent with the result in tomatoes. Apart from the resulting increase in mature fruit sugar content, silencing *SlSWEET7a* or *SlSWEET14* resulted in taller plants and larger fruits [66]. In our previous research, the content of glucose and fructose gradually increased during the fruit ripening process, accompanied by an elevation in the activity of SS, which is responsible for degrading sucrose into glucose and fructose in the cytoplasm. The decomposition of sucrose established a sucrose concentration gradient, which facilitates the continuous unloading of sucrose in the phloem [67]. Therefore, it is speculated that *VcSWEET6a* exerts a negative regulatory regulation on glucose and fructose accumulation during fruit development, while enhanced SS enzyme activity facilitates the degradation of sucrose unloaded from phloem into glucose and fructose. These two mechanisms synergistically contributed to optimizing the blueberry flavor.

## 4. Materials and Methods

### 4.1. Plant Materials

Leaves, stems, flowers, and fruits at the young stage (S1), expansion stage (S2), color-turning stage (S3), and mature stage (S4) of the cultivar Draper were selected 10, 30, 60, and 80 d after full bloom as experimental materials to investigate the spatiotemporal expression of *VcSWEET* genes in blueberry (Supplemental Figure S4). All the above samples were immediately frozen in liquid nitrogen after collection and then stored at −80 °C. Three biological replicates were performed for each experiment.

### 4.2. Identification of SWEET Gene Family in Blueberry

The genome of cultivar Draper was downloaded from the COGE database (https://genomevolution.org/coge/SearchResults.pl?s=Vaccinium&p=genome) (accessed on 3 October 2015). A hidden Markov model was used to retrieve the genomes, and the online software CD-search (https://www.ncbi.nlm.nih.gov/Structure/bwrpsb/bwrpsb.cgi) (accessed on 3 September 2022). was employed to assess the domain integrity. Subsequently, Expasy (http://web.expasy.org/) (accessed on 10 September 2022). was used for predicting physicochemical properties of *VcSWEETs*. WoLF PSORT (http://wolfpsort.org/) (accessed on 10 September 2022). was employed for subcellular localization prediction. TMHMM online website (https://services.healthtech.dtu.dk/services/TMHMM-2.0/) (accessed on 10 September 2022). was utilized for transmembrane domain prediction.

### 4.3. Phylogenetic Tree Construction

The protein sequences of the screened VcSWEETs were aligned with Arabidopsis (*Arabidopsis thaliana* L.), rice *(Oryza sativa*), and grape (*Vitis vinifera* L.) SWEETs using Clustal W, and a phylogenetic tree was constructed based on the alignment of protein sequences using MEGA7.0 with the neighbor-joining (NJ) method. The evolutionary tree is visualized and beautified using iTOL (http://itol.embl.de/login.cgi) (accessed on 12 September 2022). Online software.

### 4.4. Chromosomal Distribution and Gene Synteny Analysis

Chromosomal distribution and synteny analysis of *VcSWEETs* were performed with MCScanX (https://github.com/tanghaibao/jcvi/wiki/MCscan-(Python-version)) (accessed on 15 September 2022). Subsequently, this software was used to analyze the synteny of *V. corymbosum* with *V. macrocarpon*, *A. thaliana*, *O. sativa*, and *Z. mays* and for visualization. The amino acid sequences of SWEET proteins.

### 4.5. Analysis of Conserved Motif and Conserved Structural Domains of VcSWEETs

The conserved motifs of the protein amino acid sequences in the *VcSWEETs* family were analyzed using the MEME online tool (http://memesuite.org/tools/meme) (accessed on 10 September 2022). Subsequently, the gene annotation information (GFF3) file was processed using GSDS 2.0 (http://gsds.gaolab.org/) (accessed on 12 September 2022), and visualized using TBtools software (v2.142).

### 4.6. Cis-Acting Elements Analysis of VcSWEET Gene Family

The 2000 bp promoter sequences upstream of the initial codon of *VcSWEETs* were extracted from the genomic sequence and subsequently utilized for cis-regulatory element prediction using PlantCARE (http://bioinformatics.psb.ugent.be/webtools/plantcare/html/) (accessed on 20 September 2022), results are shown in Supplementary Table S1. The predicted results were visualized using the TBtools software (v2.142).

### 4.7. RNA Extraction and Real-Time Fluorescence Quantitative Analysis (RT-qPCR)

RT-qPCR was performed to validate the expression patterns of *VcSWEETs* in different tissues and fruit development stages. Samples stored at −80 °C were rapidly ground in liquid nitrogen, and total RNA was extracted using a CTAB-based method. cDNA synthesis was performed with the TransScript^®^ All-in-One First-Strand cDNA Synthesis SuperMix for qPCR (One-Step gDNA Removal) provided by TransGen Biotech (Beijing, China). The primers were designed using Primer Express software (v3.0.1) (Supplemental Table S2). VcEIF, which exhibited stable expression in various organs and at different fruit developmental stages, was used as an endogenous reference. qPCR was performed using the PerfectStart^®^ Green qPCR Super Mix kit (TransGen Biotech, Beijing, China) on the ABI StepOnePlus™ Real-Time PCR System (ABI, Foster City, CA, USA) with the following PCR program: an initial denaturation step at 94 °C for 30 s, followed by a denaturation step at 94 °C for 5 s, and then subjected to 40 cycles of annealing at 60 °C for 30 s and extension at 95 °C for 15 s. Each reaction was repeated three times. The tissue-specific relative expression levels of VcSWEETs were calculated using the 2^−ΔCt^ method.

### 4.8. Clone of VcSWEET6a and Multiple Sequence Alignment

The CDS of *VcSWEET6a* was cloned using the primers VcSWEET6a(CDS)-F/R (Supplemental Table S2). Multiple sequence alignment and visualization of amino acid sequences were performed using Clustalw (https://www.genome.jp/tools-bin/clustalw) (accessed on 11 November 2022) and ESPript (https://espript.ibcp.fr/ESPript/cgi-bin/ESPript.cgi) (accessed on 11 November 2022), respectively.

### 4.9. Subcellular Localization of VcSWEET6a Protein

To investigate the subcellular localization of the VcSWEET6a protein, the full-length cDNA of VcSWEET6a was cloned into the pBWA(V)HS vector (Supplemental Table S2). Both recombinant plasmids GFP: VcSWEET6a and empty vector pBWA(V)HS, serving as the negative control, were transformed into Agrobacterium tumefaciens GV3101 and then transferred into *N. benthamiana* leaves via Agrobacterium-mediated transient transformation. The plants were allowed to grow in darkness for 48 h, and GFP fluorescence was examined using laser confocal scanning microscopy (TCL SP8, Lecia, Wetzlar, Germay).

### 4.10. Functional Characterization of VcSWEET6a by Heterologous Expression in Yeast

To investigate the transport substrate of VcSWEET6a, the CDS of *VcSWEET6a* was cloned into the yeast expression vector pDR196 using the primers VcSWEET6a-196-F/R (Supplemental Table S2). Subsequently, it was transformed into a hexose transport-deficient yeast strain EBY.VW4000 that only grows on a selection medium containing maltose as the carbon source. As a negative control, we also transformed an empty pDR196 vector. The maltose in the SD selection medium was substituted with the respective sugar form. EBY.VW4000-positive clones were serially diluted and cultured on SD/-Ura plates supplemented with 2% (*w*/*v*) sugar as the exclusive carbon source to assess yeast growth. Yeast cultures were incubated at 30 °C for 3–5 d prior to photography.

### 4.11. Transient Overexpression of VcSWEET6a and Measurement of Sugar

The full-length cDNA of *VcSWEET6a* was cloned into the pGREENII-62-SK vector (Supplemental Table S2). Both recombinant plasmids pGREENII-62-SK:VcSWEET6a and empty vector pGREENII-62-SK, as the negative control, were transformed into Agrobacterium tumefaciens GV3101 and subsequently injected into blueberry fruit at S2 period. The fruits were then labeled, bagged, and observed for development after 48 h. Using RT-qPCR, four independent transgenic lines were verified with transgene expression. The content of fructose, glucose, and sucrose was determined using the D-Glucose Content Assay Kit, Plant Fructose Content Assay Kit, and Plant Sucrose Content Assay Kit provided by BOXBIO. The experiments were conducted in triplicate. Relative gene transcript levels were analyzed using analysis of variance (ANOVA) to determine significant differences between the expression levels using the GraphPad Prism 8. Tukey’s test was used to determine significant differences among the genes at each time point.

## 5. Conclusions

In summary, we conducted a genome-wide analysis of the *SWEET* gene family in blueberry and predicted their potential roles in plant development. Particularly, we focused on the role of *VcSWEET6a*, which is specifically expressed in fruit and exhibited a downregulated pattern with the development and maturation of fruit. ER-located VcSWEET6a specifically transported glucose and fructose. An increase in sugar concentration inhibits the transport of hexose by VcSWEET6a. When *VcSWEET6a* was transiently overexpressed in blueberry fruit, it exhibited negative feedback on fructose and glucose but promoted the accumulation of sucrose. Our findings will lay the foundation for further research into the role of SWEETs and improvement in the quality of blueberries.

## Data Availability

Data contained within the article.

## Supplementary Materials

The following supporting information can be downloaded at: https://www.mdpi.com/article/10.3390/ijms26031055/s1.
