# Supplementary figures and images for: Characterization of the SWEET Gene Family in Blueberry (Vaccinium corymbosum L.) and the Role of VcSWEET6 Related to Sugar Accumulation in Fruit Development
Source: Int J Mol Sci. 2025 Jan 26;26(3):1055. doi: 10.3390/ijms26031055 (PMC11817227; doi:10.3390/ijms26031055)

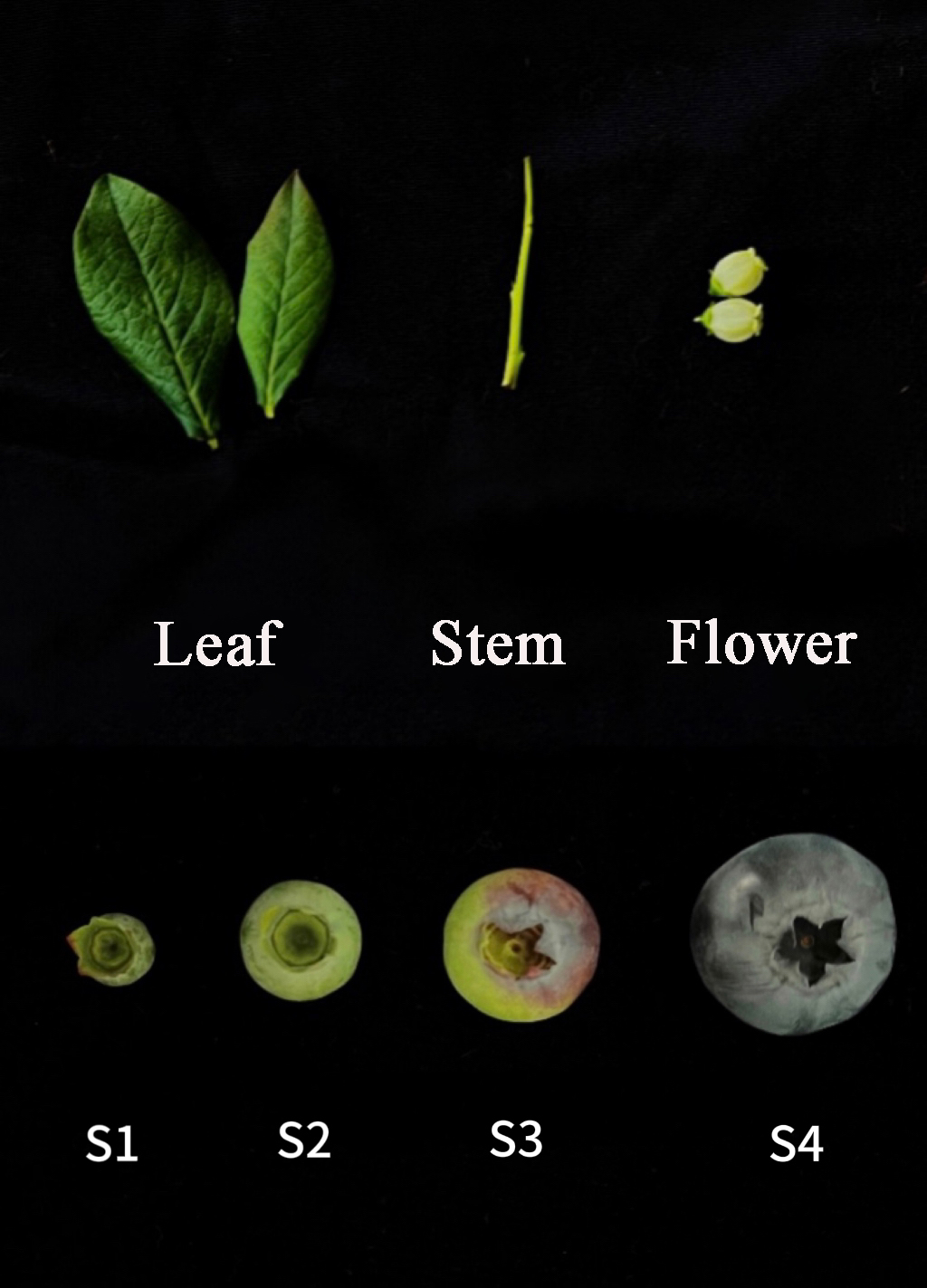

Supplement: Supplementary file 1 [file ijms-26-01055-s001.zip › Supplemental file/S4Different tissues and fruits at different developmental stages of Vaccinium corymbosum L..jpg]

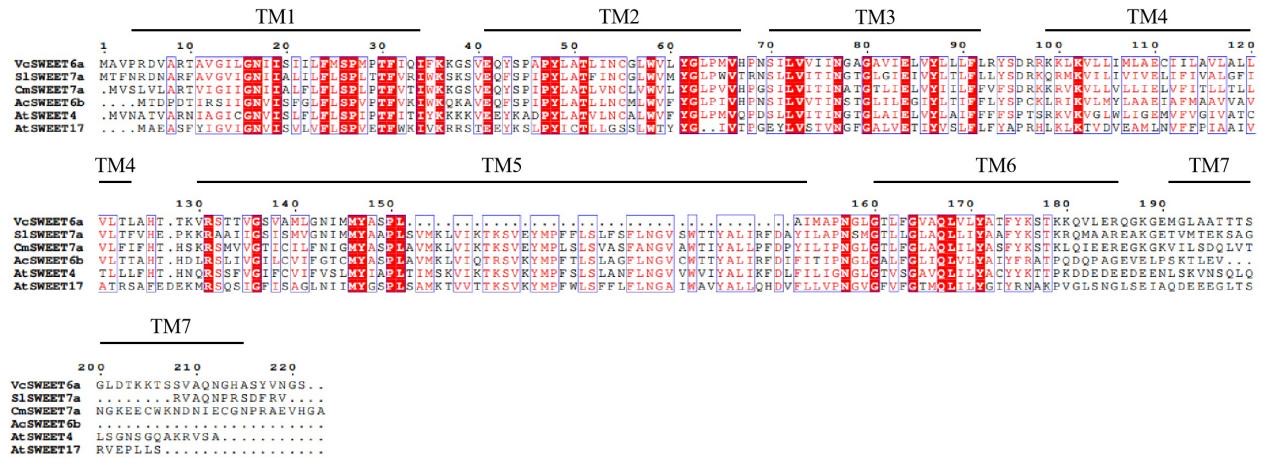

Supplement: Supplementary file 1 [file ijms-26-01055-s001.zip › Supplemental file/Supplemental figure 1. Sequence alignment of SWEET proteins..JPG]

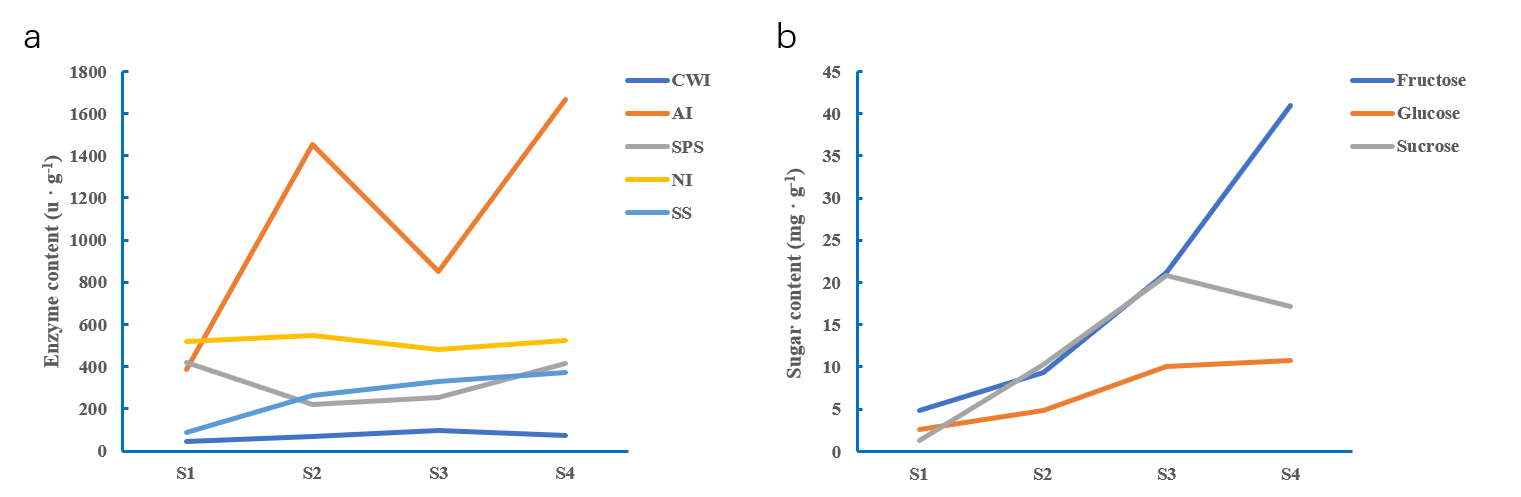

Supplement: Supplementary file 1 [file ijms-26-01055-s001.zip › Supplemental file/Supplemental figure 2. The sugar content and related enzyme activities in the blueberry fruit as the fruit matures..png]

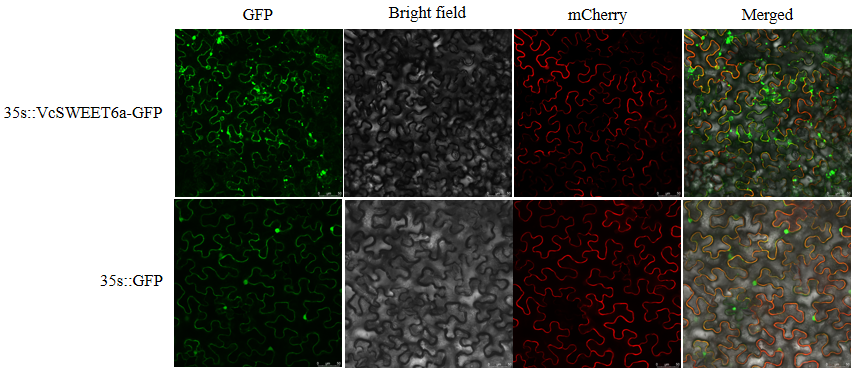

Supplement: Supplementary file 1 [file ijms-26-01055-s001.zip › Supplemental file/Supplemental figure 3. Subcellular location analysis of VcSWEET6a was performed in N. benthamiana leaf..png]
